# COVID-19 Testing Behavior as a Predictor of COVID-19 Vaccination in Southeastern Louisiana: A Longitudinal Cohort Study

**DOI:** 10.3390/vaccines12121338

**Published:** 2024-11-27

**Authors:** Sara Al-Dahir, Saba Barri, Klaus Heyer, Ashley M. Taylor, Ala’a Khalil, Mohamed Belkhouche, Ibrahim Hamed, Liliana Cosenza, Malack Jwayyed, Malaak Saad, Tina Gerard, Leslie S. Craig, Daniel F. Sarpong, Daniel Salmon

**Affiliations:** 1 College of Pharmacy, Xavier University of Louisiana, New Orleans, LA 70125, USA; sbarri@xula.edu (S.B.); amtaylor@xula.edu (A.M.T.); ihamed@xula.edu (I.H.); malackjwayyed@gmail.com (M.J.); 2 Elaine P Nunez Community College, Chalmette, LA 70043, USA; kheyer@nunez.edu (K.H.); akhalil@nunez.edu (A.K.); abelkhouche@gmail.com (M.B.); 3 Independent Researcher, New Orleans, LA 70119, USA; liliana.cosenza@gmail.com; 4 Cedar Pharmacy, 5029 Veterans Memorial Blvd Suite D1, Metairie, LA 70006, USA; cedarpharmacynola@gmail.com; 5 START Corporation, Covington, LA 70433, USA; tina.gerard@startcorp.org; 6 Independent Researcher, Bridgetown BB 15001, Barbados; lcraig1@tulane.edu; 7 Office of Health Equity Research, Yale School of Medicine, Yale University, New Haven, CT 06510, USA; daniel.sarpong@yale.edu; 8 Department of International Health, Bloomberg School of Public Health, Johns Hopkins University, Baltimore, MD 21205, USA; daslmon1@jhu.edu

**Keywords:** vaccination strategies, vaccine hesitancy, COVID-19 testing, health education

## Abstract

**Background:** Global COVID-19 vaccination effort faces the challenges of vaccine hesitancy and resistance, rooted in misinformation and institutional distrust. Addressing these barriers with customized messaging is essential, yet the relationship between vaccine hesitancy and other health-seeking behaviors, like COVID-19 testing, has been underexplored. **Method:** This study assessed COVID-19 vaccine uptake in Southeastern Louisiana across 10 pharmacies and clinics in areas with historically high rates of COVID-19 infection. Using a longitudinal cohort design from Fall 2022 through Fall 2023, a total of 377 participants from diverse backgrounds were surveyed while seeking routine care at partner organizations. Baseline data was collected on demographics, vaccine knowledge, attitudes, and test-seeking behaviors. Information on COVID-19 testing and vaccination were self-reported and verified, as applicable, in the patient’s medical records. All data was analyzed using descriptive statistics, log-binomial to yield risk ratios, and an ordinal logistic regression for vaccine series completion. **Results:** Among the 377 participants, 207 were unvaccinated while 170 received the vaccine. Among the unvaccinated individuals, 53 received a half-dose, 97 a full dose, and 14 a booster. Notably, 75% of unvaccinated and 89% of vaccinated participants underwent COVID-19 testing. Individuals who were tested were 1.71 times more likely to be vaccinated (95% CI 1.03, 2.84), while previous vaccine refusal was associated with lower vaccine acceptance (0.77; 95% CI 0.54, 1.09). In the bivariate and multivariate analysis, COVID-19 testing behavior was positively associated with COVID-19 vaccine uptake. **Conclusions:** Exploring the connection between COVID-19 testing and vaccination provides valuable insights for future public health messaging to mitigate vaccine hesitancy.

## 1. Introduction

Vaccines, as a cornerstone of healthcare interventions, have worked to prevent and curb the spread of infectious diseases [1]. The Centers for Disease Control and Prevention (CDC) reports that COVID-19 vaccinations are significantly associated with reducing an individual’s risk of severe illness, hospitalization, and death [2]. However, some regions in the United States, particularly the Southern United States, still face challenges with vaccine hesitancy and vaccine refusal, which continue to drive COVID-19 health disparities [3]. As of September 2022, the Louisiana Department of Health (LDH) reported that 59% of the population had initiated the original COVID-19 vaccination, and 53% had completed the primary vaccine series [4]. Although these numbers outperformed forecasted vaccine uptake in Louisiana, they do not reach forecasted community vaccination needs and reflect one of the lowest vaccination rates in the country compared to several states with >75% completing the initial vaccination series [5].

Evidence suggests that COVID-19 testing plays a significant role in shaping public attitudes toward vaccination, with studies showing that individuals engaged in regular COVID-19 testing were more likely to accept vaccines due to increase awareness of the virus’s prevalence and their own vulnerability [6,7]. Testing also provided direct experiences with the healthcare system, which could strengthen trust and compliance with vaccination efforts [8]. Moreover, behavioral science research suggests that individuals who were frequently tested for COVID-19 perceived higher risks from the virus, thus increasing their likelihood of getting vaccinated [9]. These findings underscore the importance of integrating testing campaigns with vaccination efforts to enhance public health outcomes in future pandemics [10,11]. Additionally, targeted communication strategies that highlight the connection between testing and the importance of vaccination can mitigate hesitancy and encourage widespread vaccine uptake [12].

Yet, while previous studies have explored factors influencing COVID-19 vaccine uptake [13,14], few studies have coupled addressing testing and vaccination behaviors [15], and a significant gap remains in assessing whether individuals who undergo COVID-19 testing are more likely to receive the vaccine. This study aims to address this gap by investigating associations between testing and vaccination behaviors. By examining this relationship, we aim to determine if COVID-19 testing serves as a motivator for vaccine uptake. This may contribute valuable insights to the ongoing efforts to promote vaccination against the virus. This study employed a novel interventional approach: delivering COVID-19 vaccine education at the point of prescription pick up/drop off at pharmacies and during health visits for clinics and pharmacies. The study uniquely identifies COVID-19 testing as a predictor of vaccine uptake, providing a new perspective on the relationship between testing behavior and vaccination. Unlike prior studies, which focused mostly on vaccine hesitancy, this research links regular healthcare engagement through testing to increased vaccine acceptance.

## 2. Materials and Methods

### 2.1. Study Design and Participants

This study employed a pre- and post-test survey design across three interview points with patients seeking routine care within pharmacies and clinics employing a purposive sampling technique. This mixed-methods longitudinal cohort study occurred between September 2022 and October 2023. This data collection was conducted at 10 locations across Southeastern Louisiana, with preferential sampling in areas with higher rates of COVID-19 infection. These locations were selected based on their geographic proximity and representation of diverse populations within the target region. Sites were recruited based upon patient demographic characteristics, including the proportion of patients who were African American or from rural or lower socioeconomic backgrounds.

### 2.2. Sample Size Calculation and Recruitment

The sample size calculation used the following assumptions: (1) two-tailed significance level of 0.05; (2) statistical power of 80%; (3) a conservative baseline vaccine completion rate of 50% to be increased to 65% (15% difference) after the intervention; and (4) demographic breakdown of 65% African American, 10% Hispanic, and 20% rural. Fifteen-percent was chosen as the incremental difference to reflect vaccine initiator proportions to reflect the median vaccine numbers reported across the United States as of Fall 2022. These parameters yielded the required sample size of 375 participants, assuming a 10% loss to follow-up over the 12-month intervention period. A total of 377 participants were recruited, with increased recruitment from rural, Hispanic, African American, or socioeconomically disadvantaged backgrounds. Socioeconomic disadvantage was defined based on self-reported challenges, such as participation in SNAP benefits, housing insecurity, and enrollment in state and federal benefit programs. Regional diversity was achieved by recruiting individuals from suburban, urban, and rural areas. Caucasian participants were selected from socioeconomically disadvantaged or rural/semi-rural backgrounds to identify clusters of vaccine-hesitant individuals. Preferential sampling occurred from zip codes with a median income of $38,423 per year, which is the median income for Orleans parish [16]. All participants were confirmed patients of the healthcare facility partners and included those seeking COVID-19 testing or routine care. The healthcare facility partners included three independent community pharmacies, five federally qualified health centers, and two private community clinics. Participants were selected based on specific eligibility criteria, including incomplete vaccination status, active engagement with healthcare services (e.g., seeking COVID-19 testing or routine care), and demographic characteristics that ensured representation of diverse racial, ethnic, and socioeconomic groups. Recruitment focused on individuals from clinics and pharmacies located in areas with health disparities. The study was approved by Xavier University of Louisiana’s Institutional Review Board, study IRB#850. We obtained informed consent from all participants and gave them the option to withdraw at any time.

### 2.3. Data Collection

Data collection for this study involved a structured baseline survey, which was administered to participants to gather demographic information and insights into their professional experience. Baseline surveys were collected between September 2022 and March 2023, with midpoint and follow-up occurring within 12 months of the baseline survey. Data from baseline survey data collection are reported here as a comprehensive assessment of the participants’ knowledge, attitudes, and concerns regarding COVID-19 testing and vaccination. Self-reported participant information, including vaccination and COVID-19 testing, was confirmed for accuracy via cross-referencing the electronic health records in the clinic or pharmacy. Information regarding the recruitment site (i.e., clinic vs. pharmacy) and reason for visit (i.e., wellness/medical counseling/sickness vs. prescription vs. vaccination) was documented. In the case of COVID-19 testing, if not present in the medical record, the patient’s self-report was also accepted. Survey questions also captured key demographic variables, such as age, race, ethnicity, gender, insurance ownership, area of residence, and educational background. Economic disadvantage was assessed via single (yes/no) responses to 10 questions on past or current experience of financial challenges (e.g., Myself or my family receive SNAP Benefits (Food Stamps); I am homeless). Endorsement of any of the nine economic disadvantage questions (vs. selection of the final response option, “None of the above apply to me”) was coded as economic disadvantage. Patients self-reported if they had ever received any type of COVID-19 test prior to the baseline survey. In addition to demographic data, the survey assessed participants’ knowledge, attitudes, and concerns related to vaccine delivery, with a specific focus on the COVID-19 vaccine and COVID-19 testing. Participants were asked about prior experiences with COVID-19 testing, infection, and vaccination (as applicable), their perceptions of the personal and public health importance of COVID-19, and any concerns regarding vaccination. Vaccine hesitancy was assessed via employing the World Health Organization Vaccine Hesitancy Scale. Participants responded via indicating the level of agreement with five statements (i.e., Vaccines are necessary to protect the health of adults; Vaccines do a good job in preventing the diseases they are intended to prevent; If I do not get vaccinated, I may get sick with the disease; If I do not get a vaccine, I may spread the disease to someone else; Vaccines are very safe) measured on a five-point Likert scale (Cronbach’s alpha = 0.79), which was later converted to tertiles based upon distribution. Vaccine hesitancy scores were transformed and categorized as not hesitant, neutral, or most hesitant.

All data were collected electronically via the digital survey platform, Qualtrics© [17], ensuring efficient data entry and management. Vaccination status was confirmed in the patient’s medical record and via the statewide vaccine system, Links© [18]. This approach allowed for the systematic collection of quantitative data, which was essential for understanding the factors influencing participants’ perceptions and decisions related to vaccine delivery.

### 2.4. Data Analysis

The data collected through the digital survey platform, Qualtrics© were exported into a secure database and was analyzed using STATA version 14.2 [19]. Descriptive statistics, such as frequencies, proportions, means, and standard deviations, were calculated to summarize the demographic characteristics, vaccine uptake, and reasons for vaccine acceptance or refusal. Chi-square tests or Fisher’s exact tests were used to assess associations between demographic variables and vaccine behaviors. The primary outcome variable was dichotomized to having received any COVID-19 vaccine since introduction (vs. receiving no COVID-19 vaccine) while the secondary outcome used an ordered variable to explore completion of the vaccine series (i.e., 0—no vaccine, 1—incomplete primary series, 2—completed primary series, and 3—received at least 1 eligible booster after primary series completion). A completed primary series was defined as one Johnson-Johnson or two Pfizer©/Moderna© vaccines. The final model, employing a generalized linear model (GLM) with a binomial family and a log link function and best-fit prediction with AIC and BIC, was used to render risk ratios (RRs) and 95% confidence intervals (CIs) around the primary vaccination outcome. Variables were included in the final model prediction if they were significant in the bivariate analysis or if the literature recommended inclusion based upon significance in other studies, such as sex at birth and race. The final best fit model was based upon AIC and BIC values rendered. As the completion of the vaccine series could be ranked from none to completed with a booster, an ordinal logistic regression explored factors associated with the secondary vaccination completion.

## 3. Results

The baseline characteristics of the study participants are described in Table 1, for the full sample (*n* = 377), and by COVID-19 testing behaviors. Differences in baseline characteristics were noted in the site of patient recruitment (clinic vs. pharmacy), if the participant knew anyone who had COVID-19 infection, that person’s health outcome, and their education level (high school or less vs. post-high school). Otherwise, the participants were similar based on whether they had a history of having a COVID-19 test or not.

Differences in COVID-19 vaccine behaviors across several demographic characteristics, COVID-19 testing exposure, infection experiences, and vaccination concerns were then examined (Table 2). Of the participants, 170 (45.1%) individuals reported receiving at least 1 vaccine, and 207 (54.9%) were never vaccinated. In the binary analysis, individuals who had received a COVID-19 test were 2.02 times (95% CI 1.31, 3.09) as likely to have initiated a COVID-19 vaccine versus those who had not received a COVID-19 test. Notably, a high percentage of vaccinated individuals (89%) had undergone COVID testing in the past, while a substantial percentage of unvaccinated individuals (75%) had also received a COVID test in the past.

Within the overall population (*n* = 377), it was observed that 153 individuals had a previous history of refusing any vaccine in the past that was offered to them, comprising 106 out of 207 patients (51.2%) in the non-vaccinated group and 47 out of 170 patients (27.6%) in the vaccinated group. The patients who had previously refused any vaccine were 0.546 times (95% CI 0.43, 0.73) as likely to have received at least one COVID-19 vaccine. The patients who had received a COVID-19 diagnosis were 1.01 times (95% CI 0.81, 1.26) as likely to receive a COVID-19 vaccine versus those with no previous COVID-19 diagnosis. Individuals with higher educational attainment were more inclined to accept COVID-19 vaccination, with 85 out of 207 patients in the non-vaccinated group and 91 out of 170 patients in the vaccinated group having a completed high school education or higher, 1.32 (95% CI 1.05, 1.64). Increasing vaccine hesitancy was associated with the decreasing likelihood of having received at least one COVID-19 vaccine, with a decrease in vaccine uptake as vaccine hesitancy increasing from moderately hesitant at 0.63 (95% CI 0.45, 0.87) to most hesitant at 0.25 (95% CI 0.15, 0.41) as likely to receive a COVID-19 vaccine compared to the lowest tertile of vaccine-hesitant individuals.

Unadjusted and adjusted risk ratios across multiple predictive models are presented in Table 3. The primary predictor variable, receiving a COVID-19 test in the past, are presented as unadjusted bivariate and adjusted risk ratios in all of the models. In all model analysis, the primary predictor variable, COVID-19 testing, was significant and positively associated with COVID-19 vaccination. A final best-fit model is presented in Table 4. Several factors were associated with the likelihood of COVID-19 vaccine uptake. Individuals with concerns about serious vaccine side effects, such as blood clots and death, exhibited a lower likelihood of COVID-19 vaccination (RR = 0.56, *p* = 0.008). Those visiting clinics or pharmacies were more likely to have received the COVID-19 vaccine (RR = 1.42, *p* = 0.038). Greater hesitancy (i.e., higher vaccine hesitancy scores) were significantly associated with a reduced likelihood of COVID-19 vaccination among those with the highest level of vaccine hesitancy (RR = 0.35, *p* < 0.001). Older individuals had a higher likelihood of COVID-19 vaccination compared to their younger counterparts (RR = 1.25), while individuals with a history of vaccine refusal exhibited a decreased likelihood of COVID-19 vaccination (RR = 0.77), although neither was significant in the final model Additionally, no significant associations were observed between COVID-19 vaccination status and the participant’s race or education level. Participants who underwent COVID-19 testing displayed an increased likelihood of receiving the COVID-19 vaccine (RR = 1.71, *p* = 0.038).

In the overall vaccinated population (164), among those vaccinated, 53 (32.3%) had an incomplete primary vaccine, 97 (59.1%) had a complete primary series, and 14 (8.5%) had a complete primary series and had received at least 1 booster shot (Table 5). COVID-19 testing was a significant predictor of vaccination status among those who completed the primary series and those who also received one additional booster (OR 2.61 and 4.33, respectively).

## 4. Discussion

The main predictor variable, COVID-19 testing, was significantly associated with initiation of the COVID-19 vaccination uptake and completion in this Southeastern Louisiana sample of participants from clinics and pharmacies. Reports in the literature of associations between COVID-19 vaccine hesitancy and COVID-19 testing are mixed, with some studies reporting no associations between vaccine hesitancy and previous positive COVID-19 test [5] while others finding that having been tested for COVID-19 was associated with a lower likelihood of being “not at all likely” to vaccinate [20]. Notably, findings from the trend analysis in this study indicate that individuals who participated in COVID-19 testing also had higher rates of vaccination across all three opportunities of the vaccine series. Testing behavior is likely to facilitate the vaccine uptake through several mechanisms. First, individuals who undergo testing are already engaged with the healthcare system, creating opportunities for trust-building and vaccine education. Second, testing increases personal risk awareness, which may motivate proactive health behaviors such as vaccination. These mechanisms align with behavioral theories emphasizing the role of repeated exposure and healthcare interaction in promoting preventive actions. This suggests that test seeking may also serve as an important opportunity to offer the vaccine at the point of care.

Drivers of COVID-19 testing behaviors include convenience, invasiveness of the testing method, affordability, and location of the administration of the COVID-19 test [21,22]. The availability of rapid testing may lead to an increase in testing rates in spite of a delay in receiving test results [23]. COVID-19 testing behaviors were often correlated with the consequences of positive test results, such as the need to self-isolate and the reduction of income due to employment policies around a positive COVID-19 test [24,25]. Previous infection and severity of infection have been associated with a willingness for symptom-driven testing [26]. In this study, previous infection status was not associated with vaccination likelihood. Proximity to individuals with a COVID-19 infection and the severity of that infection were associated with testing behavior but not associated with vaccination uptake. Drivers of COVID-19 disparities centered around race, education, living in rural areas, and healthcare trust [5,10,11]. In this study, in both the bivariate and multivariate analysis, these factors were not associated with health seeking with regards to testing or preventative health strategies, such as COVID-10 vaccination. In this post-pandemic period, disparities in testing access have already begun to emerge [27].

The ending of the Public Health Emergency Declaration (PHED) has led to a disproportionate financial impact of prevention and care seeking among underserved populations [28]. Creative solutions to keeping populations engaged in self-directed testing and care have emerged as a response to a systems-focused approach to surveillance [29]. Inconsistent testing data can lead to a poorly informed public health response [30]. Further, COVID-19 testing barriers lead to under-reporting in COVID-19 surveillance data on a global scale [31]. Expiration of the PHED has led to a shift in the sentinel surveillance framework [32]. Thus, consideration for personal motivations to seeking testing and care in symptom positive or exposed individuals will be critical to providing continued data on COVID-19 [28]. High-resolution information on vaccine acceptance can inform policies to achieve population immunity against COVID-19, while state-level diagnosis rates, reported in surveys and supported by state surveillance data, provide important insights into the pandemic’s impact at a regional level [33].

Consistent with other studies, this study demonstrated that COVID-19 vaccine refusal was not an isolated behavior. Instead, COVID-19 vaccine refusal was a continuation of previous vaccine hesitancy. Patients who had refused any vaccine in the past, particularly the influenza vaccine, were more likely to refuse a COVID-19 vaccine. Individuals with a previous history of any vaccine refusal were 0.77 (95% CI 0.54, 1.09) times as likely to receive a COVID-19 vaccine. These findings substantiate the notion that patients who have previously declined a vaccine are more likely to do so in the future. Vaccination initiation and completion are associated with a conglomerate of health behaviors and access. Demographic and social factors have been previously associated with higher acceptance of testing and greater vaccination likelihood, while offering incentives like lotteries and rewards made little difference in areas where people were already hesitant or resistant to the COVID vaccine [33,34,35,36,37,38]. Several mechanisms have been employed to efficiently address these barriers, and interventions focused upon adaptable messaging tailored to specific local contexts and communities are needed [13,39].

As anticipated, most patients in our study were acquainted with someone who had contracted COVID-19. There were more patients in the non-vaccinated group who knew someone who had contracted the virus prior to getting the vaccine and after getting the vaccine. Infection after vaccination may be a driver of the lack of subsequent vaccination, although this variable did not populate in the final model. Further, this study suggests that the social impact of COVID-19 illness severity played a role in COVID-19 vaccine perceptions and uptake. There were more patients in the non-vaccinated cohort who were acquainted with individuals who had contracted COVID-19 and recuperated without complications. This circumstance might lead a patient to surmise that they could undergo a similar course of recovery if they were to contract the virus. Moreover, the severity of COVID-19 infection appears to be a factor influencing vaccine adoption. This perception could potentially lead them to believe that vaccination is unnecessary.

Institutional distrust, misinformation, and existing skepticism about vaccines highlight the complex nature of vaccine behavior, which goes beyond the simple “pro-vax” or “anti-vax” label [3,4,39,40,41]. The way that vaccination safety messages are framed can greatly influence vaccine intent, and public health messaging must be careful as messaging is pivotal to improving vaccine uptake [23]. Approaches beyond targeted messaging should focus on healthcare worker-driven dialogue for specific groups with an emphasis on enhancing vaccine knowledge [13]. Yet, mistrust in healthcare providers may also impede vaccine adoption in a given population. Given that this study was conducted in the Southern region of the United States, which has a historically documented record of race-based disparities, healthcare mistrust among this population was anticipated [42].

COVID-19 testing has been coupled with simultaneous respiratory illness testing strategies to lead to streamlined stewardship of both testing and treatment [43]. To that end, post-pandemic treatment strategies continue to include multiple testing modalities, pre-emptive vaccination at the beginning of the respiratory illness season, and post-infection therapies to decrease the duration and severity of illness [44,45]. Though these programmatic approaches provide insight for a healthcare infrastructure response to the post-pandemic COVID-19 period, additional information on behaviors related to both COVID-19 testing and COVID-19 vaccination are still required.

The study does have limitations. Although the study did reach a large number of people, a larger sample size would have allowed a more granular analysis of demographic predictors of vaccination via a stratified approach. Although the Southern region of Louisiana does have some level of homogeneity of experience, there are differences with regards to access when comparing rural and urban/suburban participants. Another aspect that warrants further investigation pertains to the higher representation of economically disadvantaged patients in the non-vaccinated group when compared to the vaccinated group. Understanding the reasons behind this disparity is an area that merits more extensive exploration. In Southeastern Louisiana, all the recruitment sites offered testing and vaccination during the COVID-19 testing and vaccine roll-out periods. Although testing and vaccine access varied throughout the state of Louisiana, within the recruitment area, these sites reported frequent patient utilization for these services prior to the research study. These sites were located in primarily urban, suburban, and semi-rural areas. Thus, generalizability to rural areas and areas within limited healthcare access is also limited.

This cohort study has established a direct association between vaccine uptake and disease testing, particularly among patients with a history of vaccine refusal. These findings underscore the importance of understanding past behaviors and attitudes toward vaccines when designing interventions aimed at increasing vaccine uptake. The persistence of hesitancy among those who have previously declined vaccination highlights the need for targeted educational efforts and trust-building initiatives, especially in communities with historical mistrust of the healthcare system.

## 5. Conclusions

This study delved into the enduring obstacle of vaccine hesitancy, revealing a strong connection between individual’s past vaccine behaviors and their current choices regarding COVID-19 vaccination. By specifically addressing the gap in the literature, this study explored whether individuals who undergo COVID-19 testing are more inclined to subsequently receive the vaccine. The findings suggest that COVID-19 testing may indeed serve as a motivator for vaccination, providing valuable insights into strategies for promoting vaccine uptake. One potential mitigating factor could be reinforcing COVID-19 vaccine messaging through multiple trusted sources [46]. Additionally, the study identified that testing behavior plays a role in influencing the decision to get vaccinated. To effectively tackle the challenge of vaccine uptake, it is important not only to provide education but also to engage patients in comprehensive conversations about COVID-19 at multiple points of care seeking across healthcare setting, including pharmacies and clinics. Despite the observed association between testing behavior and vaccine uptake, several barriers to vaccine acceptance remain unaddressed, including mistrust in healthcare systems, misinformation, and logistical challenges, such as transportation and clinic availability. These factors likely influenced our results and limit the generalizability of the findings to populations with different socioeconomic or healthcare access conditions. Further research should investigate tailored interventions to overcome these barriers.

In general, addressing vaccine hesitancy and resistance requires a multifaceted approach that considers the complexity of the problem, the diverse reasons for hesitancy across different populations, and the crucial role of healthcare professionals. This study’s findings on the relationship between COVID-19 testing and vaccine uptake contribute valuable insights to ongoing public health efforts, underscoring the need for integrated strategies that encompass testing, education, and outreach. This study recognizes that increasing vaccine uptake in vulnerable communities is not solely dependent on vaccine availability but also on effective education and outreach efforts.

## Figures and Tables

**Table 1:** Baseline characteristics of participants based upon exposure and COVID-19 testing behaviors.

**Covariates**	**Received a** **COVID-19 Test in the Past** **(*n* = 308)**	**Did Not Receive a COVID-19 Test in the Past** **(*n* =69)**	**Risk Ratio (95%CI) (*p*-Value)**
	Frequency and Percentage		
**Age**			
17–49 (ref)	222 (72%)	55 (80%)	
50 and Above	86 (28%)	14 (20%)	1.07 (0.97, 1.18) 0.16
**Race**			
African American (ref)	119 (39%)	31 (45%)	---
Caucasian	142 (46%)	28 (41%)	1.05 (0.95, 1.17) 0.338
Other	47 (15%)	10 (14%)	1.04 (0.89, 1.20) 0.602
**Ethnicity**			
Non-Hispanic (ref)	270 (88%)	60 (87%)	---
Hispanic	38 (12%)	9 (13%)	0.98 (0.85, 1.15) 0.875
**Sex at Birth**			
Male (ref)	113 (37%)	30 (43%)	---
Female	195 (63%)	39 (57%)	1.05 (0.95, 1.17) 0.308
**Insurance**			
None (ref)	29 (9%)	13 (19%)	---
Medicare/Medicaid	209 (68%)	44 (64%)	1.2 (0.97, 1.48) 0.095
Private	70 (23%)	12 (17%)	1.24 (0.99, 1.54) 0.060
**Area of Residence**			
Rural/Semirural (Rural) (ref)	84 (27%)	15 (21%)	---
Suburban/Urban	224 (73%)	54 (79%)	0.95 (0.86, 1.05) 0.317
**Education**			
High School or Less (ref)	155 (50%)	46 (67%)	---
More Than High School	153 (50%)	23 (33%)	1.13 (1.03, 1.24) 0.013
**Have You Experienced Any Economic Disadvantages in the Past 12 Months**			
No (ref)	96 (31%)	20 (29%)	---
Yes	212 (69%)	49 (71%)	0.98 (0.887, 1.09) 0.718
**Recruitment Site**			
Clinic (ref)	210 (68%)	57 (83%)	---
Pharmacy	98 (32%)	12 (17%)	1.13 (1.03, 1.24) 0.007
**Visit Reason**			
Wellness/Med Counsel/Sickness (ref)	224 (73%)	54 (77%)	---
Prescription	62 (20%)	11 (16%)	1.05 (0.94, 1.18) 0.392
Vaccination	22 (7%)	5 (7%)	1.01 (0.84, 1.22) 0.937
**Have You Ever Had a COVID-19 Diagnosis**			
No (ref)	60 (34%)	9 (4%)	---
Yes	116 (66%)	192 (96%)	1.45 (1.30, 1.62) 0.000
**Which Side Effect Are You Concerned About From the COVID-19 Vaccine?**			
None (ref)	157 (51%)	38 (56%)	---
Nonserious Side Effects	49 (16%)	8 (12%)	1.07 (0.94, 1.21) 0.306
Serious Side Effects	102 (33%)	23 (33%)	1.02 (0.91, 1.13) 0.738
**Do You Know Anyone Who Had a COVID-19 Infection?**			
No (ref)	52 (17%)	23 (33%)	---
Yes	256 (83%)	47 (67%)	1.22 (1.04, 1.43) 0.013
**Did Anyone You Know Have** **COVID-19 After Getting the** **COVID-19 Vaccine?**			
No (ref)	82 (32%)	20 (43%)	---
Yes	174 (68%)	26 (57%)	1.08 (0.97, 1.21) 0.159
**Among People Who You Know Who Had COVID-19, What Happened to That Person?**			
Recovered (ref)	172 (69%)	33 (77%)	---
Hospitalized	31 (12%)	7 (16%)	0.972 (0.83, 1.14) 0.735
Passed Away	46 (18%)	3 (7%)	1.12 (1.02, 1.23) 0.018
**Have You Ever Refused a Vaccine?**			
No (ref)	185 (60%)	39 (57%)	---
Yes	123 (40%)	30 (43%)	0.973(0.88, 1.07) 0.592
**Vaccine Hesitancy Score (Tertiles)**			
Not Hesitant	184 (60%)	37 (54%)	---
Moderately Hesitant	55 (18%)	12 (17%)	0.99 (0.87, 1.12) 0.827
Most Hesitant	69 (22%)	20 (29%)	0.93 (0.82, 1.06) 0.269

RR—risk ratio; CI—confidence interval.

**Table 2:** Predictors of COVID-19 Vaccine Completion Among Study Participants and Risk Ratios.

**Covariates**	**Did Not Receive a COVID-19** **Vaccine (*n* = 207)**	**Received at Least One COVID-19** **Vaccine (*n* = 170)**	**RR (95% CI) (*p*-Value)**
	Frequency and Percentage		
**Have You Been Tested for COVID-19?**			
No (ref)	52 (25%)	17 (11%)	---
Yes	155 (75%)	153 (89%)	2.02 (1.31, 3.09) 0.001
**Age**			
17–49 (ref)	172 (83%)	105 (62%)	---
50 and above	35 (17%)	65 (38%)	1.71 (1.39, 2.11) <0.001
**Race**			
African American (ref)	78 (38%)	72 (42%)	---
Caucasian	96 (46%)	74 (44%)	0.91 (0.71, 1.15) 0.422
Other	33 (16%)	24 (14%)	0.88 (0.62, 1.24) 0.459
**Ethnicity**			
Non-Hispanic (ref)	182 (88%)	148 (87%)	---
Hispanic	25 (12%)	22 (13%)	1.04 (0.75, 1.45) 0.798
Sex at Birth			
Male (ref)	76 (37%)	67 (39%)	---
Female	131 (63%)	103 (61%)	0.939 (0.75, 1.18) 0.589
**Insurance**			
None (ref)	19 (9%)	23 (14%)	---
Medicare/Medicaid	149 (72%)	104 (61%)	0.75 (0.55, 1.03) 0.072
Private	39 (19%)	43 (25%)	0.96 (0.68, 1.35) 0.805
**Area of Residence**			
Rural/Semirural (ref)	61 (29%)	38 (22%)	---
Suburban/Urban	146 (71%)	132 (78%)	1.24 (0.94, 1.63) 0.134
**Education**			
High School or Less (ref)	122 (59%)	79 (46%)	---
High School GRADUATE and Above	85 (41%)	91 (54%)	1.32 (1.05, 1.64) 0.016
**Have You Experienced Any Economic Disadvantages in the Past 12 Months**			
No (ref)	58 (28%)	58 (34%)	---
Yes	149 (72%)	112 (66%)	0.86 (0.68, 1.08) 0.192
**Recruitment Site**			
Clinic (ref)	169 (82%)	98 (58%)	---
Pharmacy	38 (18%)	72 (42%)	1.78 (1.45, 2.2) <0.001
**Visit Reason**			
Wellness/Med counsel/Sickness (ref)	170 (82%)	107 (63%)	---
Prescription	27 (13%)	46 (27%)	1.63 (1.30, 2.05) <0.001
Vaccination	10 (5%)	17 (10%)	1.63 (1.18, 2.26) 0.003
**Have you ever had a COVID-19 diagnosis?**			
No (ref)	97 (47%)	79 (46%)	---
Yes	110 (53%)	91 (54%)	1.01 (0.81, 1.26) 0.94
**Which side effect are you concerned about experiencing from the COVID-19 vaccine?**			
None (ref)	100 (48%)	95 (56%)	---
Nonserious Side Effects	11 (5%)	46 (27%)	1.66 (1.37, 2.01) <0.001
Serious Side Effects	96 (46%)	29 (17%)	0.48 (0.34, 0.68) <0.001
**Do you know anyone who had the COVID-19 infection?**			
No (ref)	40 (19%)	35 (21%)	---
Yes	167 (81%)	135 (79%)	0.96 (0.73, 1.26) 0.757
**Did anyone you know have COVID-19 after getting the vaccine?**			
No (ref)	58 (35%)	44 (33%)	--
Yes	109 (65%)	91 (67%)	1.05 (0.81, 1.38) 0.698
**Among people who you know who had COVID-19, what happened to that** **person?**			
Recovered (ref)	119 (73%)	86 (67%)	--
Hospitalized	19 (12%)	19 (15%)	1.19 (0.84, 1.7) 0.334
Passed	26 (16%)	23 (18%)	1.12 (0.79, 1.57) 0.515
**Have you ever refused a vaccine?**			
No (ref)	101 (49%)	123 (72%)	--
Yes	106 (51%)	47 (28%)	0.56 (0.43, 0.73) <0.001
**Vaccine Hesitancy Score (Tertiles)**			
Not Hesitant (ref)	89 (43%)	132 (78%)	--
Moderately Hesitant	42 (21%)	25 (15%)	0.63 (0.45, 0.87) 0.004
Most Hesitant	76 (36%)	13 (8%)	0.25 (0.15, 0.41) <0.001

RR—risk ratio; CI—confidence interval.

**Table 3:** Multivariate analysis of the impact of receiving a COVID-19 test in the past on having initiated a COVID-19 vaccine.

	RR	95% CI *p*-Value
Unadjusted (Participant Received a COVID-19 Test in the Past)	2.02	(1.22, 3.33) 0.006
Adjusted (Participant Received a COVID-19 Test in the Past)		
Concerned About COVID-19 Vaccine Side Effects; Reason for Visit to Clinic or Pharmacy; Vaccine Hesitancy Score; Refused a Vaccine the Past; Age and Race	1.86	(1.1, 3.02) 0.02
Concerned About COVID-19 Vaccine Side Effects; Reason for Visit to Clinic or Pharmacy; Vaccine Hesitancy Score; Refused a Vaccine the Past Age; Race, and Education	1.82	(1.10, 3.02) 0.02
Concerned About COVID-19 Vaccine Side Effects; Recruitment Site; Vaccine Hesitancy Score; Refused a Vaccine in the Past; Age, Race, and Education	1.71	(1.03, 2.84) 0.038
Concerned About COVID-19 Vaccine Side Effects; Reason for Visit to Clinic or Pharmacy; Vaccine Hesitancy Score; Refused a Vaccine the Past; Age, Gender, Race, and Education	1.72	(1.04, 2.85) 0.036
Know Some Who Had a COVID-19 Infection; Concerned About COVID-19 Vaccine Side Effects; Reason for Visit to Clinic or Pharmacy; Vaccine Hesitancy Score; Refused a Vaccine in the Past; Age, Gender, Race, and Education	1.72	(1.04, 2.87) 0.036
Know Some Who Had a COVID-19 Infection: Concerned About COVID-19 Vaccine Side Effects; Reason for Visit to Clinic or Pharmacy; Vaccine Hesitancy Score; Refused a Vaccine in the Past; Age, Gender, Race, Education, and Area of Residence.	1.74	(1.04, 2.89) 0.034

RR—risk ratio; 95% CI—confidence interval; *p*-value.

**Table 4:** Final Model Variable for the Impact of COVID-19 testing behavior on the outcome of COVID-19 vaccine uptake.

Variable	RR	95% CI *p*-Value
Have You Ever Received a COVID-19 Test (Yes)	1.71	(1.03, 2.84) 0.038
Are You Concerned About Side Effects From the COVID-19 Vaccine?		
No	---	---
Nonserious Side Effects	1.29	(0.89, 1.86) 0.17
Serious Side Effects	0.56	(0.34, 0.86) 0.008
Recruitment Site (Pharmacy)	1.42	(1.02, 1.99) 0.038
Vaccine Hesitancy Score		
Not Hesitant	---	---
Moderately Hesitant	0.81	(0.52, 1.27) 0.354
Most Hesitant	0.35	(0.19, 0.63) 0.001
Have You Ever Refused a Vaccine That You Were Offered in the Past? (Yes)	0.77	(0.54, 1.09) 0.143
Age Greater Than 50 Years Old	1.25	(0.90, 1.73) 0.181
Race (African American or Other)		
African American	---	---
Caucasian	1.15	(0.81, 1.64) 0.441
Other	1.06	(0.66, 1.71) 0.799
Education Greater Than High School	1.24	(0.91, 1.69) 0.174

RR—risk ratio; 95% CI—confidence interval; *p*-value.

**Table 5:** Characteristics of Participants Based Upon COVID Vaccine Status Completion and COVID-19 Testing.

**Covariates**	**None (*n* = 208)**	**Received 1 of 2 of the Primary Vaccine Series (*n* = 53)**	**Completed the** **Primary Vaccine Series (*n* = 97)**	**Completed the Primary Vaccine Series and** **Received 1 Eligible** **Booster** **(*n* = 14)**
	Frequency (Percentage), OR (95% CI), *p*-value			
COVID Test				
Yes	156 (75%)	48 (91%)	86 (89%)	13 (93%)
No (ref)	52 (25%)	5 (9%)	11 (11%)	1 (7%)
	--	**3.2 (1.21, 8.47)** 0.019	**2.61 (1.29, 5.26)** 0.007	**4.33 (0.553, 33.93)** 0.163

## Data Availability

Requests for data sets may be accessible by directly contact the researcher, Sara Al-Dahir (saaldah@xula.edu).
